# Sleep–Wake Cycle in Young and Older Mice

**DOI:** 10.3389/fnsys.2019.00051

**Published:** 2019-09-24

**Authors:** Sara Soltani, Sylvain Chauvette, Olga Bukhtiyarova, Jean-Marc Lina, Jonathan Dubé, Josée Seigneur, Julie Carrier, Igor Timofeev

**Affiliations:** ^1^Department of Psychiatry and Neuroscience, Faculty of Medicine, Université Laval, Québec, QC, Canada; ^2^CERVO Brain Research Centre, Québec, QC, Canada; ^3^Center for Advanced Research in Sleep Medicine, Centre Intégré Universitaire de Santé et de Services Sociaux du Nord-de-l’Ile de Montréal, Montreal, QC, Canada; ^4^École de Technologie Supérieure, Montreal, QC, Canada; ^5^Department of Psychology, Université de Montréal, Montreal, QC, Canada

**Keywords:** sleep–wake cycle, LFP, delta power, SWS, REM, wake, sleep fragmentation

## Abstract

Sleep plays a key role in multiple cognitive functions and sleep pattern changes with aging. Human studies revealed that aging decreases sleep efficiency and reduces the total sleep time, the time spent in slow-wave sleep (SWS), and the delta power (1–4 Hz) during sleep; however, some studies of sleep and aging in mice reported opposing results. The aim of our work is to estimate how features of sleep–wake state in mice during aging could correspond to age-dependent changes observed in human. In this study, we investigated the sleep/wake cycle in young (3 months old) and older (12 months old) C57BL/6 mice using local-field potentials (LFPs). We found that older adult mice sleep more than young ones but only during the dark phase of sleep-wake cycle. Sleep fragmentation and sleep during the active phase (dark phase of cycle), homologous to naps, were higher in older mice. Older mice show a higher delta power in frontal cortex, which was accompanied with similar trend for age differences in slow wave density. We also investigated regional specificity of sleep–wake electrographic activities and found that globally posterior regions of the cortex show more rapid eye movement (REM) sleep whereas somatosensory cortex displays more often SWS patterns. Our results indicate that the effects of aging on the sleep–wake activities in mice occur mainly during the dark phase and the electrode location strongly influence the state detection. Despite some differences in sleep–wake cycle during aging between human and mice, some features of mice sleep share similarity with human sleep during aging.

## Introduction

Sleep patterns change throughout life. In humans, the highest density of slow waves moves from the occipital cortex in preschoolers to centro-parietal areas during adolescence, and to frontal areas in adulthood (Kurth et al., 2010). Middle-age subjects show a reduced sleep efficiency, duration, slow-wave density, and a reduced amplitude of slow waves compared to young adults (Carrier et al., 2011; Mander et al., 2017). However, mice studies showed conflicting results. A study compared EEG recordings above the somatosensory cortex of 6 months old to 18–24 months old C57BL/6JOlaHsd male mice and found an increase in the sleep amount in the older group as well as an increase in the delta range power (Panagiotou et al., 2017). Another group found that C57BL/6 mice show only a non-significant trend for an increase in their daily amount of non-REM sleep from 3 to 6 to 12 months old, however showing a significant decline at 2 years old compared to 1 year old (Hasan et al., 2012).

Another study performed in C57BL/6J mice with LFP recordings from motor and visual cortices in 4 months and a half, 1 year old, and 2 years old mice showed a linear increase in the total sleep time with age (McKillop et al., 2018). The same study also showed that the amplitude of slow waves was larger in the younger group compared to the two older groups, but the slow wave incidence was only significantly increased in the 2 years old group (McKillop et al., 2018). It was also demonstrated that the increase in total sleep in aging mice depend on their inability to maintain prolonged wakefulness (Wimmer et al., 2013).

As different mice lines show different sleep patterns and are differently affected by age (Franken et al., 1998; Hasan et al., 2012), it becomes important to compare results of studies performed in the same mouse line.

Sleep was long thought to be a global state; however, it was shown both in humans (Nir et al., 2011) and in rats (Vyazovskiy et al., 2011) that sleep can be local. A study performed in Long Evans rats showed a high congruence of slow-wave sleep (SWS) using frontal and parietal EEGs as well as mPFC and hippocampal LFPs; however, congruence was lower for REM sleep and even much lower during transition states (Durán et al., 2018). A study conducted in C57BL/6 mice concluded that slow waves occur regularly during REM sleep especially in primary sensory and motor cortices, and mainly in layer 4 (Funk et al., 2016). A vast majority of cortical projections in carnivores and primates, but not in rodents are patchy, local, and intracortical (Gilbert and Wiesel, 1983; Rockland, 1985; Lund et al., 1993, 1994; Schuz et al., 2006; Van Hooser et al., 2006). Such difference in corticocortical circuitry could explain regional differences in the expression of sleep in mice, specifically slow waves which should be expressed more locally throughout the cortex. While local slow waves during REM sleep were also reported in human (Bernardi et al., 2019), it would be expected that slow waves are more local in mice than in humans.

We hypothesized that the different cerebral areas reported in the above-mentioned studies done on male rodents might explain the conflicting results on aging reported in mice. We propose that mice might not be the best model of human sleep in aging, but some features will reproduce changes observed in aging humans.

Here we investigated the daily distribution of sleep–wake states, the delta power modulation, and slow waves parameters in male C57BL/6 young (3 months old) and older (12 months old) mice. We found that 1-year-old C57BL/6 mice sleep significantly more than 3 months old mice over 24 h and these differences are especially significant during the dark phase. We also report that some cerebral areas will consistently reveal less REM sleep than others as they remain in deep SWS while most areas display REM oscillations. The SWS delta power changed with aging, but these differences were topographically specific. Older mice revealed a trend of increased incidence of slow waves only in the frontal cortex. Older mice also demonstrated significant increase in sleep fragmentation. We conclude that only some features of human sleep in aging could be effectively investigated in mice.

## Materials and Methods

### Experiments and Animals

All experiments were performed in accordance with the guideline of the Canadian Council on Animal Care and approved by the Université Laval Committee on Ethics and Animal Research. Experiments were performed on young (3 months old) male C57Bl/6 mice (*n* = 8) and older (12 months old) male C57Bl/6 mice (*n* = 9) to compare the sleep characteristics of the two groups of age. We analyzed recordings for 10–25 consecutive days in each animal obtained at least 1 week after electrode implantation and beginning of continuous recording to allow animals to recover from anesthesia and to adapt to a tethered condition.

### Sterile Surgery and Electrodes Implantation

All surgeries were performed under sterile conditions. The mice were first anesthetized with 1–2% isoflurane, the head was shaved and then fixed in the stereotaxic frame. Subcutaneous injection of buprenorphine (0.1 mg/kg) was applied for analgesia and saline (0.9% NaCl) (s.c.). The incision site and all pressure points were injected with a mixture of Bupivacaine (0.25%)/Lidocaine (0.5%). The head was cleaned with three passages of chlorhexidine (0.5%) in alternation with alcohol (70%) before the skin incision. After opening the skin above the skull, three alternating passages of a bleach solution (0.03% sodium hypochlorite) and hydrogen peroxide (3%) were used to clean the skull. Small holes were drilled for the reference, anchoring screws, and LFP electrodes. We used custom-made electrodes (stainless steel wires, 125 μm diameter, Perfluoroalkoxy (PFA)-insulated) and in the majority of experiments they were implanted in three different cortical regions to a depth of 600 μm from the cortical surface: frontal cortex (AP: +2.6 mm; ML: −1.5 mm); somatosensory cortex (AP: −0.94 mm; ML: −3 mm, and AP: −1.75 mm, ML: −2 mm). In separate experiments (*n* = 2) we implanted 14 LFP electrodes (Figure 6) to obtain multisite LFP recordings from the major part of the dorsal cortical surface. One stainless steel screw over the cerebellum was used as a reference and four anchoring screws (two screws on each side of the skull) were used to secure the implant. Two 75 μm electrodes [single-stranded stainless-steel wire (PFA-insulated)], were inserted into the neck muscle to record the electromyogram (EMG) activity. All LFPs, EMG electrodes, and the reference electrode were connected to a Nano-Miniature Omnetic Connector and were covered and fixed with dental acrylic (Dentsply Canada). Mice received subcutaneous injections of Anafen (5 mg/kg) and saline (0.5–1 ml) for 2 days post-surgery.

### Recordings

The recordings were done within the standard animal facility settings. The standard mice cages were modified to enable the passage of a tethering cable connecting the animal. Miniature custom-made buffer pre-amplifier (voltage amplification coefficient 1, but it amplifies current) was attached directly to the head and it was used to reduce movement artifacts in cables (not shown). These pre-amplifiers were connected to the commercially available AM amplifiers (Model 3000, A-M system, Sequim, WA, United States). The signals were hardware band-pass filtered between 1–100 Hz (LFP) and 10–300 Hz (EMG), the notch filter was used at 60 Hz. The light–dark cycle was 12 h/12 h with the lights off at 19:00. LFPs and EMG were continuously recorded (sampling rate 1 kHz) with LabChart (AD instruments, Colorado Springs, CO, United States) 24 h per day for at least 3 weeks. The recordings started at the end of the surgery; however, we analyzed only data obtained at least 5 days after the end of anesthesia.

### Automatic States of Vigilance Identification

**FIGURE 1:**
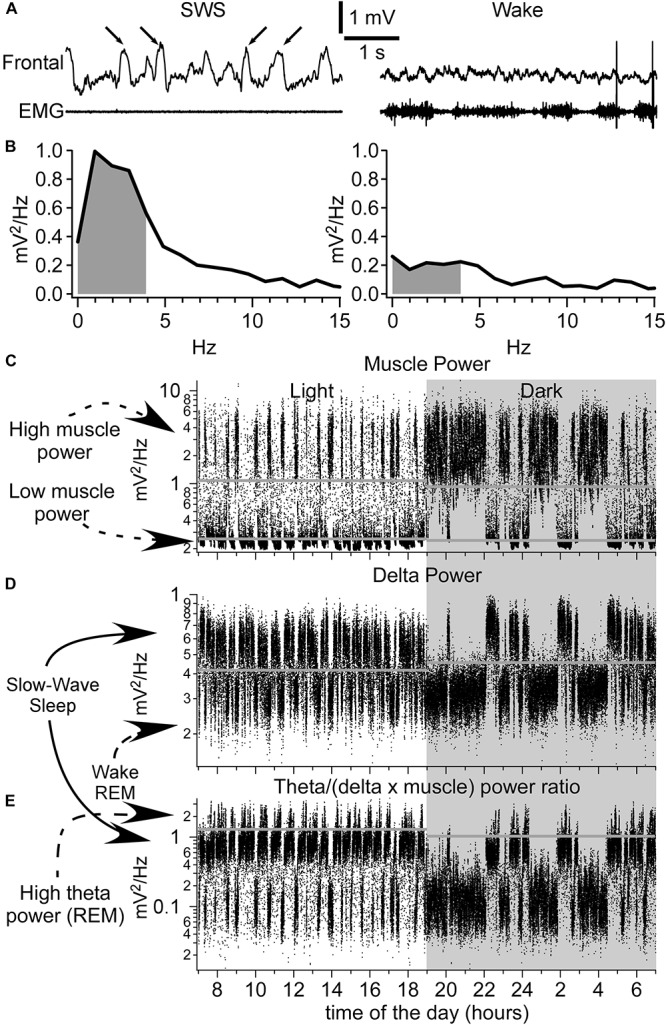
Identification of sleep–wake states. **(A)** A segment of LFP recorded from the frontal cortex and EMG (neck muscle) during slow-wave sleep (left panel, arrows indicate the slow waves) and wake (right panel). Below are the corresponding FFT analysis **(B)**. Gray indicates the area used to calculate the delta power (0.2–4 Hz). **(C)** Muscle power over 24 h. Shaded area indicates the dark period. **(D)** Delta power pattern for 24 h. Each dot represents 5 s of recordings. Note the bimodal distribution pattern of delta power with high delta power values corresponding to slow-wave sleep and low delta power values corresponding to either wake or REM sleep. **(E)** Calculated ratio over 24 h of theta power divided by the delta power and further divided by the muscle power. High ratio values correspond to REM sleep having a high theta power, and low delta and muscle powers. Horizontal gray lines correspond to threshold values set for shown examples. Note that the thresholds were different for recordings obtained during light phase vs. dark phase.

We used custom-written routine in Igor Pro (version 6, Wavemetrics, OR, United States) or MATLAB (Version R2015b) to automatically detect states of vigilances. Fast Fourier Transform (FFT) was calculated using 5 s time window for all LFP channels recorded as well as for EMG (Figure 1A). A brief comparison of the detection with two different time-windows (5 and 30 s, not shown) revealed that longer time-windows missed multiple microarousal periods (Watson et al., 2016). Therefore, we analyzed all data with 5 s windows with a sliding time window of 1 s for computing the power of activities of interest. First, we run Fourier analysis with 0.1 Hz resolution from which we obtained absolute values of Fourier amplitude. We then defined the delta power as the area between 0.2 and 4 Hz (Figure 1B), the theta power as the area between 5 and 9 Hz, and the muscle power as the area between 10 and 300 Hz. The power was initially computed in each channel, but then we realized that on multiple occasions, higher values of power of different frequencies could co-occur with lower values in other channels. To ensure the stability of state detection, in most of analysis, the delta and theta power was computed in each channel and then it was summated across channels. As the theta power alone was not always a very good indication of REM sleep (Borbély et al., 1984) and it was stronger/weaker depending on the area recorded, therefore, we divided the theta power by the delta power and also by the muscle power. This theta/(delta × muscle) ratio was very efficient to detect REM episodes as only period with low delta and low muscle tone would give high values, corresponding to REM sleep. Thresholds for state detection were set manually. Because SWS-delta power is higher during the dark period as opposed to the light period, the thresholds for the dark vs. light periods were set to a different value. Despite the use of our custom-made preamplifier, the movement artifacts could be recorded and at the level of LFP it would produce large values in delta power frequency range. Therefore, the first step was to set a threshold for muscle power, which had bimodal distribution (Figure 1C). If the muscle power was high, it was considered as wake. In the remaining segments, we set thresholds for LFP delta power, that also had bimodal distribution (Figure 1D). The high values of LFP delta power in the absence of muscle contractions were considered as SWS. The remaining segments were analyzed with the theta/(delta × muscle) ratio (Figure 1E). If it was high, we consider it as REM sleep, otherwise it was quiet wakefulness. Less than 1% of segments, typically at the transitions between states, did not fall in these criteria [very low muscle tone power, low delta power, and low theta/(delta × muscle) ratio] and we qualified them as undefined state, which was not analyzed further.

### Slow Waves Detection

The detection of slow waves was achieved by MATLAB scripts using a pattern recognition method described previously (Bukhtiyarova et al., 2016, 2019). The method allowed us to segment the original signal, preparing input data for neural network training, training the neural network to classify the chosen templates into groups and post-processing of the results. Briefly, we created a library of 8903 LFP segments obtained from nine channels of young mice and six channels of old mice. These segments were manually labeled as “SW” or “Noise.” The library was randomly divided in three parts: 60% of it served as templates to train feed-forward artificial neural network (1 hidden layer of 20 neurons and 1 output layer with 2 possible outputs for “SW” and “Noise”), 20% was used for validation, and 20% to test the quality of detection. The precision of LFP segments classification (positive predictive rate) and recall (true positive rate) for the obtained network was 86.7 and 80% correspondingly for “SW” and 96.2 and 97.7% for “Noise,” the overall accuracy was 94.8%. This network was applied for the entire dataset after its segmentation and was followed with post-processing.

The amplitude of detected slow waves was measured from zero crossing to the maximum of depth-positive wave from traces digitally re-filtered between 1 and 100 Hz. The duration of detected slow waves was measured at half amplitude of the signal digitally filtered between 1 and 4 Hz.

### Statistics

To evaluate the effect of age on different sleep parameters, linear mixed models (LMM) were computed using SPSS25 (IBM). This approach is now recommended over mean-based tests (*t*-tests) in neuroscience to resolve the issue of independence of multiple measures in a single individual by controlling individual variation in each subject (Koerner and Zhang, 2017). Each variables-of-interest was modeled using restricted maximum-likelihood method (REML) as varying according to fixed effects of interest (which will be interpreted and discussed below) and random effects (to correct for intra-individual variability). For each variable, two random factors were taken into account: (1) the intercept across all days recorded for each mouse and (2) the slope across all recorded days. An autoregressive covariance structure was specified in the model to account for covariance between timepoints. In essence, these random effects control for intra-individual variability due to data recorded in a different number of days between mice. Convergence and significance of each model were required before interpreting results. Models converged and were significant (*p* < 0.001) for all variables of interest.

Relative duration (%) of each state was averaged for each recorded hour. Two independent two-way LMMs were computed to model the hourly relative duration of each state (one model per period: light and dark). These models accounted for random effects, modeled *hour* as a repeated fixed effect (12 h) and *age* as an independent fixed effect (*two age group*). Then, relative duration (%) of each state of vigilance (wake, SWS, REM) was averaged over 24 h, dark and light periods, and modeled in independent LMMs with specified random effects and *age* as a fixed effect (*two age groups*).

Two independent three-way LMMs were computed to model hourly delta power in different channels (one model per period: light and dark). These models accounted for random effects, modeled *hour* and *electrode* as repeated fixed effects (*12 hours x 3 electrodes*) and *age* as an independent fixed effect (*two age groups*). In addition, delta power in SWS was also averaged over 24 h, dark and light periods, and modeled in independent two-way LMMs with random effects, *age* as a fixed effect (two age groups), and *electrode* as a repeated fixed effect (*three electrodes*). Finally, slow wave characteristics (density, amplitude, and duration) were analyzed with two-way LMMs specifying *age* as a fixed effect (*two age group*) and *electrode* as a repeated effect (three electrodes).

Interaction terms between all fixed factors were always included in each model. When interaction terms were significant at *p* < 0.05, they were decomposed with simple effect tests reported in relevant figures. If not, main effects were interpreted and are reported in figures.

### Histology

The mice were deeply anesthetized with ketamine/xylazine and transcardially perfused with saline followed by 4% paraformaldehyde (PFA). The brains were kept in the PFA solution overnight and were cut coronally in 50-μm-thick slice with a vibrotome in PBS solution or after 2 days in a 30% sucrose solution with a microtome. Sections were treated with Nissl staining for the electrode location visualization.

## Results

### State of Vigilance Distribution Over 24 H

**FIGURE 2:**
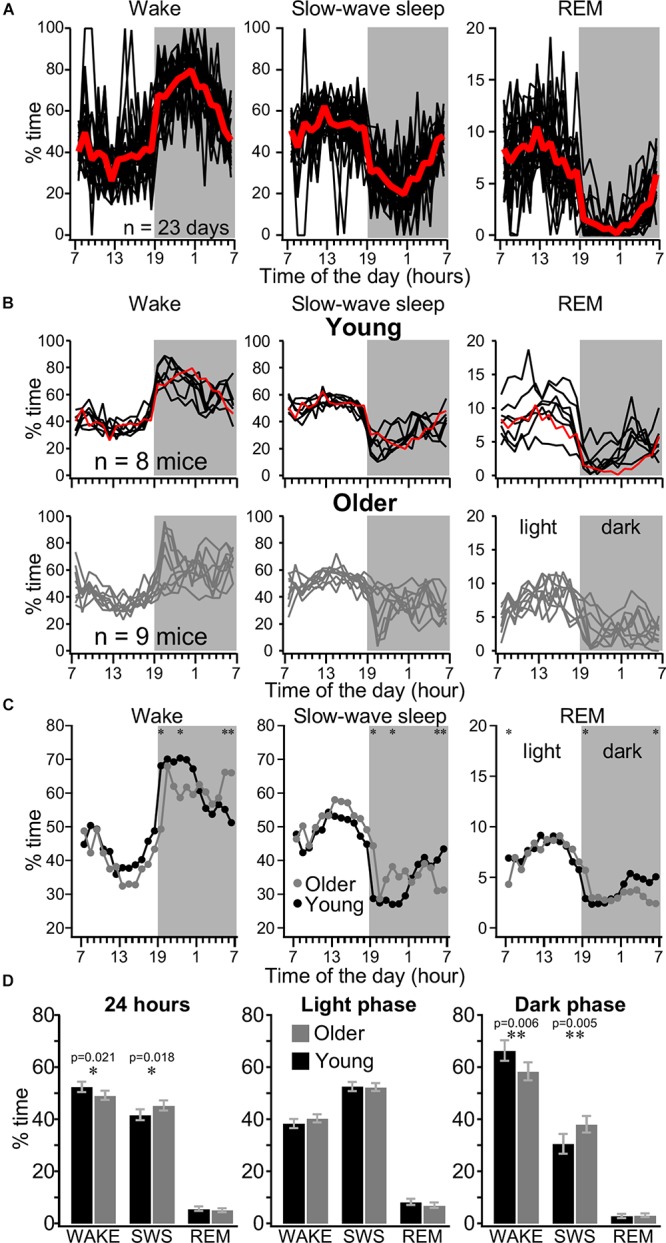
Distribution of sleep–wake states in young and older mice over 24 h. **(A)** Distribution of wake, SWS, and REM sleep recorded over 23 consecutive days with 1-h resolution in individual mice (black lines) and their average (red line). **(B)** Distribution of wake, SWS, and REM sleep in young (upper panels) and older (lower panels) mice. Each line represents averaged values of one animal over several days of recordings, the red line is an average from the example mice shown in panel **(A)**. **(C)** Hourly distribution of states of vigilance for 24 h for young (black circles, *n* = 99 days from eight mice) and older (gray circles, *n* = 67 days from nine mice) mice. Shaded area indicates the dark phase. Printed values are estimated marginal means computed in six independent mixed linear models (one for each state of vigilance and phase of light–dark cycle) with age and hour factors. **(D)** 24 h and light/dark cycle distribution of states of vigilance. Printed values are estimated marginal means from independent mixed linear models. Note that older mice show more SWS than young mice over a day, but this difference occurs mainly during the dark phase. Significant statistical differences in each hour are indicated by asterisk, ^∗^*p* < 0.05, ^∗∗^*p* < 0.01. Error bars indicate the 95% confidence interval.

With rare exceptions, the same animal had similar daily distribution of sleep–wake states with more sleep during the light period and more wake during the dark period of the dark–light cycle. Figure 2A shows a distribution of automatically detected states over 23 consecutive days in one mouse implanted with electrodes at the age of 3 months. Periodical absence of sleep in 1-h segments during light phase was mainly due to environmental factors, such as unusual activities within or around the recording room rather than a natural behavior of mice. We examined the performance of our automatic method of state detection. On six arbitrarily chosen 4-h long segments, the agreement of automatic detection against experienced user detection was 92.59 ± 2.97% (not shown). The major disagreement was in the detection of very brief states (typically microarousals) which were often not detected (not considered) by an experienced observer.

We analyzed the automatically detected state distribution for 99 days from eight young mice and 67 days from nine older mice. Out of at least 3 weeks of recording per animal, we typically analyzed 12–14 days from each animal, but up to 23 days, starting from the second week after electrode implantation and connection to recording cables. Some days were skipped because of occasional technical difficulties. The same animal typically demonstrated very similar sleep–wake pattern across days (Figure 2A) except occasional long wake states during the day, likely due to unusual environmental conditions (atypical noise in animal facilities). We compared the hourly distribution of state of vigilance for 24 h and the overall distribution of the states in 1 day as well as the sleep pattern according to the light/dark cycle in young and older mice (Figures 2B–D). As expected, young mice were more awake than older mice at the beginning of the dark phase of cycle (60–80% of time) compared to the light phase of the light/dark cycle and with the progression of the dark phase of cycle the sleep proportion increased. The older mice had a more variable sleep–wake behavior. Out of nine older mice, five had 70–95% of wake at the beginning of the dark phase of cycle, but the four other animals had <50% of wake at the same time period (Figure 2B). LMM analysis showed the presence of a significant interaction between hour and age for wake time in the dark phase (*p* < 0.05). Simple effects analyses show that young mice were more awake at the beginning of the dark phase of the light/dark cycle (Figure 2C), while the older mice were more awake during morning time (at end of the dark phase of cycle). A significant interaction between age and hour was also found for SWS and REM in the dark phase of the light/dark cycle (*p* < 0.05). Simple effect analysis demonstrate that older mice had more sleep than young at the beginning of the dark phase of the light/dark cycle while old had less sleep than young at the end of the dark phase (Figure 2C). For the light phase, significant interactions between age and hour were found for wake and REM (*p* < 0.05), but not for SWS. *Post hoc* tests in the light phase showed that old animals had less REM at the beginning of the cycle. Significance was not reached at any time points for *post hoc* of age effects on wake during light phase. Finally, main effect of age on SWS duration was non-significant in the light phase. Overall, older mice had more SWS than young mice and these differences were due to longer time spent in SWS during the dark phase of the light/dark cycle (Figure 2D). Overall, these results suggest that differences in sleep-wake behavior of young vs. older mice depend largely on the specific period of the dark–light cycle.

**FIGURE 3:**
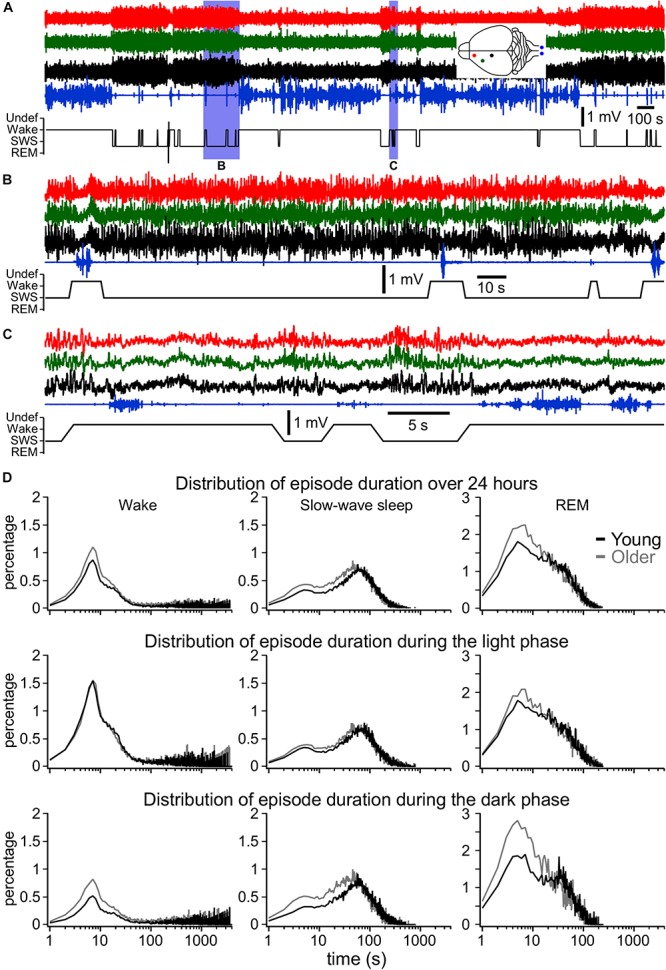
Fragmentation of sleep and wake states. **(A)** One-hour long LFP recordings at the beginning of the dark phase (19:00–20:00 h) in a 1-year old mouse. **(B,C)** Expended fragments from this recording demonstrating the presence of very short wake episodes during SWS [activated LFP and increased muscle tone, **(B)**] and sleep episodes during wake [slow-wave activity and reduced muscle tone, **(C)**]. **(D)** Share of all episodes from all investigated animals and days of wake, SWS, and REM sleep of different duration with 1 s resolution for 24 h, for light phase and dark phase as indicated for young and older mice. These distributions show that older mice have larger number of short-lasting states, in particular during the dark phase.

Most states in mice were short. As it can be seen from Figures 3A–C, at the beginning of the dark phase of cycle, when the sleep pressure is low, the animal continuously switched between SWS and waking states. The SWS was often interrupted by brief waking episodes characterized by an activated LFP in all channels and increased muscle tone (Figure 3B) as well as during wake, we often observed episodes of reduced muscle tone and high power of LFP activities in the low-frequency bands, characteristic for SWS (Figure 3C). Using 5 s resolution for state detection, very brief muscle twitches (typically less than a second), even accompanied with activated LFPs were not detected as wake (not shown). During REM sleep, the LFP was typically activated and the muscle tone was mostly absent with the exception of occasional twitches. As a mean, the older mice had more states per day (2002 ± 510.5, *n* = 55 days) than young mice (1659 ± 219.9, *n* = 79 days; *p* = 0.0003, Mann–Whitney *U*-test). The results show that except wake during the light phase of cycle (*p* = 0.3615) and REM sleep during light phase of cycle (*p* = 0.2057) on a 24-h scale, the older mice had more periods of wake, SWS, and REM (*p* = 0.002, *p* = 0.0002, and *p* = 0.0237, correspondingly). During the dark phase of the dark/light cycle these differences were highly significant (all *p* < 0.0001) indicating that the states of vigilance in older mice were less consolidated compared to young mice. Overall bimodal distribution of SWS suggests that SWS episodes lasting 10 s and less represent some separate states and can be considered as microsleep states (Figure 3D). For the wake, the short episodes had likely two dominant durations with a clear maximum around 7 s and less obvious around 20 s (Figure 3D). It is possible that these two types of electrophysiological microarousal states represent some different behavioral states.

### Daily SWS Delta Power Dynamics Is Different in Young and Older Mice

**FIGURE 4:**
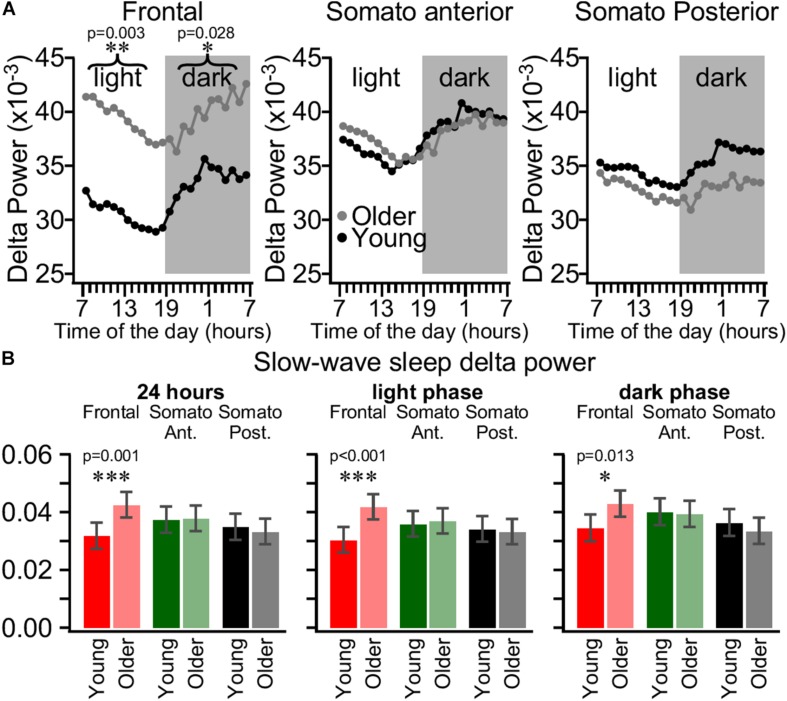
Area-specific daily SWS delta power dynamics is different in young and older mice. **(A)** Estimated mean delta power in SWS at each hour of the day for the frontal electrode (left panel), the somatosensory anterior electrode (middle panel), and somatosensory posterior electrode (right panel) for young (black circles, *n* = 99 days from eight mice) and older mice (gray circles, *n* = 67 days from nine mice). Printed values are estimated marginal means computed in two independent mixed linear models (one per light or dark phase) using factors of age, electrode, and time of the day. **(B)** Mean SWS delta power in frontal cortex (red), somatosensory anterior cortex (green), and somatosensory posterior cortex (black) for 24 h (left panel), light phase (middle panel), and dark phase (right panel) for young (bright colors) and older (light colors) mice. Error bars indicate the 95% confidence interval. Significant statistical differences are indicated by asterisk, ^∗^*p* < 0.05, ^∗∗^*p* < 0.01, ^*⁣∗∗^*p* < 0.001.

During SWS, cortical neurons have a bi-stable behavior alternating between active and silent states and during REM sleep or wake, the membrane potential has a unimodal distribution (Steriade et al., 2001; Timofeev et al., 2001). The LFP/EEG signal is essentially generated by synchronous activities of cortical neurons (Contreras and Steriade, 1995; Chauvette et al., 2010). Therefore, during wake or REM sleep, the ratio of activities below and above 4 Hz (delta range) is nearly 1, but during SWS the activity in the delta range power is six times higher than the faster frequencies power, reflecting the bi-stable behavior of cortical neurons (Mukovski et al., 2007). In our experiments, the overall delta power distribution was bimodal, with low values corresponding to waking state and REM sleep and high values corresponding to SWS (Figure 1D). The origin of delta activity during wake or REM sleep is unclear; therefore, we further analyzed the delta power dynamics only during SWS. Previous experiments on cats demonstrated that intracellular sleep slow wave activity had different amplitude in different cortical areas (Chauvette et al., 2011). Therefore, we calculated the mean hourly delta power (0.2–4 Hz) values during SWS from the frontal, somatosensory anterior, and somatosensory posterior channels and computed for the entire day with a 5 s time window sliding by 1 s (Figure 4). As expected from sleep homeostasis process (Borbely, 1982; Borbely et al., 1989), in both groups of age, the delta power in all three investigated cortical areas declined gradually during the light period followed by gradual increase in the dark phase of the light/dark cycle in the hourly distribution (Figure 4A). LMM analysis did not show a three-way interaction between age, electrode, and time, but we do report the presence of a two-way interaction (*p* < 0.05) between age and electrode in both dark and light period, demonstrating that age changes in delta power are topographically specific but constant across the time of measurement in each light/dark cycle. Moreover, *post hoc* tests showed that older mice have a significantly higher delta power in the frontal cortex, while there was no difference in the somatosensory anterior and posterior cortex for both light and dark phases. That was true both when calculated hourly (Figure 4A) and for the whole light/dark cycle or separately for the light and the dark phases (Figure 4B). As it is clearly seen in Figure 4A, in older mice, the overall delta power was the highest in the frontal cortex, lower in anterior somatosensory, and even lower in posterior somatosensory cortices, while in young mice the delta power was the lowest in the frontal cortex and it was higher in both anterior and posterior somatosensory cortex which is the opposite of what is seen in human (Carrier et al., 2011).

### Slow-Wave Features in Young and Older Mice

**FIGURE 5:**
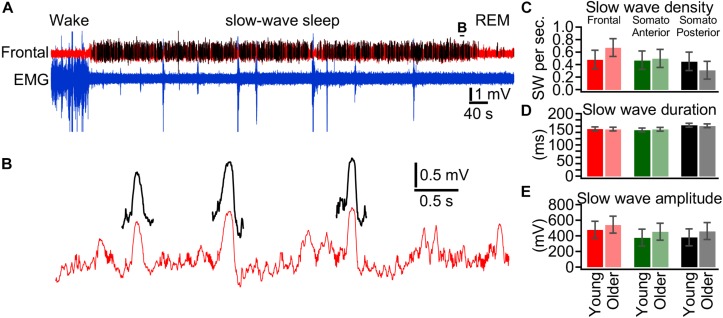
Slow wave features in young and older mice. **(A)** Segment of LFP recording from the frontal cortex (red trace) and EMG from neck muscle (blue trace). Slow waves are detected using a neural network approach and detected slow waves are depicted by black traces. **(B)** Expanded segment from **(A)** as indicated. **(C)** Slow wave density detected during slow-wave sleep over a 24 h period for young (bright colors, *n* = 18 days from nine mice) and older (light colors, *n* = 18 days from eight mice). **(D)** Duration of detected slow waves, **(E)** Amplitude of detected slow waves.

The differences in SWS delta power in different ages and areas are likely mediated either by the number of waves or by their properties. Therefore, using a neural network approach (Bukhtiyarova et al., 2019), we extracted (Figures 5A,B) and quantified individual slow waves for 18 days from seven mice in each group and in the three different cortical areas investigated (Figure 5). Using LMM, a significant interaction between age and electrode for slow wave density was found (*p* = 0.05), which suggest that age effect vary depending on the topography. Follow up *post hoc* tests show that slow wave density seems higher in old mice compared to young mice specifically in the frontal channel (*p* = 0.067, Figure 5C), while no age-difference approached significance for other channels (Figure 5C). The differences were not significant for slow wave duration or amplitude. These results suggest that a higher incidence of slow waves was likely a factor of the increase in the delta power observed in the frontal cortex of older mice.

### Area Specificity for the Expression of Sleep–Wake States

**FIGURE 6:**
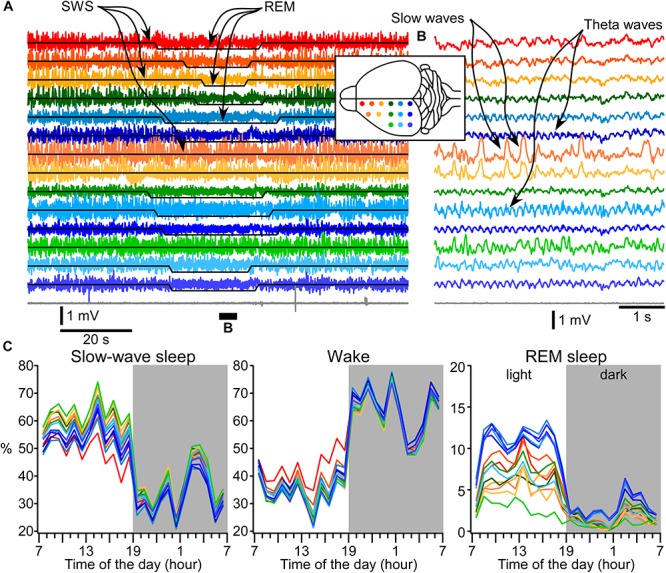
Area-specific distribution of electrographic brain states. **(A)** A segment of 14 LFP channels and muscle (gray) recordings during SWS, REM sleep, and again SWS. Signals from electrodes are color coded and the location of electrodes is indicated in the inserted drawing. **(B)** A short segment that was overall qualified as REM sleep, but two fronto-laterally located electrodes show clear slow-wave activity. **(C)** 24-h distribution of states detected based on muscle activity and each electrode individually.

It is very common that the detection of states of vigilance in rodents is done based on just one or two electrodes. Using EEG recordings in mice, it was recently shown that spontaneous sleep (Fernandez et al., 2017) or sleep deprivation differentially modulates slow wave vs. fast activities in various cortical areas (Kim et al., 2017). Combined with our data on area-specific differences in delta power, these results suggest that various areas can have different propensities for sleep and wake states. Therefore, we investigated LFP activities in 14 different cortical locations together with neck muscle EMG in intermediate age mice (6 months old, Figure 6). The overall idea of these experiments was to identify the given state of vigilance based on each LFP electrode separately and muscle activities, and then to compare the coincidence of state detection. Based on a formal criteria of state detection (Figure 1) we found that (a) the transition between states does not occur simultaneously and even in closely located areas, the delays of state transition could take up to 20 s (compare the first and second green traces, Figure 6A); (b) while REM sleep (high intensity theta rhythm and low muscle tone) can be detected in a large number of cortical areas, some channels display slow-wave activities at the same time (Figure 6B). Using formal criteria of state identification, we often observed simultaneously the presence of two and sometimes three different states in different parts of neocortex (Supplementary Movie S1). Comparison of states (two mice, 3 days per mice) shows that during the light phase the frontal cortex spends only about 50% of time in the SWS, and in somatosensory cortex, the SWS occupies about 65% of time. The differences are even more dramatic for REM sleep, which takes 12–13% of light phase in visual and retrosplenial areas and only about 3% in lateral somatosensory area (Figure 6C). Therefore, electrographic activities corresponding to SWS, REM sleep, or wake can co-occur in different cortical areas.

## Discussion

In this study we show that age effects on sleep depend on specific period of the light/dark phases. In the light phase, 3-months old and 1-year-old mice spent similar time in sleep and wake states except that 1-year-old mice spent significantly less time in REM sleep at the beginning of each period. Similarly, 1-year-old mice spent less time in REM sleep at the end of the dark phase of the cycle. Generally, in the dark phase, older mice spent globally less time in wake and more time in SWS. The increase in the total sleep duration was mainly due to an increase in the number of short (less than a minute) sleep episodes during the dark phase. The LFP delta power had remarkable regional specificity. Older mice showed larger LFP delta power in the frontal cortex compared to young mice, which can be explained by a tendency for an increase in slow wave density. We also investigated regional specificity of sleep–wake electrographic activities and found that using formal criteria, posterior regions of the cortex show more REM sleep activities (high theta power during low muscle tone), while somatosensory cortex displays more often SWS patterns.

### How Long Do States Last?

In agreement with multiple previous investigations, our study shows that overall, mice spent more time sleeping during the light part of the light–dark cycle and more time in waking state during the dark phase of the cycle (Tobler et al., 1997; Franken et al., 1999; El Helou et al., 2013). It is commonly used to call short states of activated EEG/LFP accompanied with an increase in the muscle tone in rodents as microarousals (Watson et al., 2016). However, the formal definition of microstates is unclear. Typically, in human, the state is arbitrarily defined as a stable pattern lasting >15 s (Iber et al., 2007). However, recent advances in automatic methods of state detection demonstrated that the conventional states can be much more fragmented than it was previously assumed (Koch et al., 2019). It is known that brain states in animals undergo rapid changes, which are associated with marked changes in neuronal activities in different structures (Bezdudnaya et al., 2006; Reimer et al., 2016). Human studies indicate that 500 ms is the minimal time to mediate conscious perception (Libet et al., 1967; Edelman, 2003). An essential condition for a conscious state to be generated is spatio-temporally coordinated neuronal firing (Konorski, 1967). The presence of EEG slow wave activities is a key factor for the loss of consciousness (Purdon et al., 2013; Mukamel et al., 2014) because it interrupts firing. Sleep or anesthesia-induced slow waves in cats and those measured intracellularly are mediated by neuronal hyperpolarization and silence that lasts 150–300 ms (Timofeev et al., 2001; Volgushev et al., 2006; Chauvette et al., 2010, 2011). In sleeping rats and mice, the LFP slow waves or reduced/abolished firing associated with slow waves are variable in duration, and they typically last >100 ms (Vyazovskiy et al., 2009; Bukhtiyarova et al., 2019). Therefore, the active states during sleep and anesthesia last typically longer than 500 ms and the animals are still unconscious. Thus, the minimal duration of an arousal should be longer than 500 ms and likely longer than 1 s in order to be considered as a state. On the contrary, the presence of local slow waves in some spatially restricted parts of the cortex alters behavioral responses (Vyazovskiy et al., 2011) suggesting that locally synchronized silent periods, lasting a few hundreds of milliseconds, could be considered as a state. Our study demonstrates the presence of a large number of events that follow a lognormal distribution and were identified as wake or SWS that last <10 s (Figure 3D). This suggests that they represent isolated states, which likely can be called microarousal or microsleep states. Similar activities exceeding this time likely represent either short or long states, not microstates. As our detection method occasionally leads to undefined states at state transitions (see the section “Materials and Methods”), we cannot exclude that a significant part of detected short states are due to the detection method itself. Because the delta power varied not only between cycles, but also between the beginning and the end of the cycle, and we used the same threshold throughout each phase (light or dark), we could artificially increase the detection of short states. However, there are no reasons to believe that the method would affect more the results of one group in particular; therefore, our observation of a higher number of short-lasting states (wake or SWS) in older mice compared to young mice is very solid. The state duration in mice living in natural environment could be different. For example, it is unlikely that mice foraging in the forest at the beginning of the dark phase of the sleep–wake cycle will have brief sleep episodes.

### Delta Power Differences

The anterior somatosensory electrode in our study was located in the barrel field and we did not observe age-specific differences in the sleep delta power in young vs. older mice (Figure 4). This suggests that there are very little age-dependent changes in the most important sensory system of mice. While no differences were found in the posterior somatosensory cortex (trunk area), there was an increase in delta power and a trend for an increased slow wave density in frontal cortex of older mice. Increase in delta power in frontal cortex in older mice are opposed to what is seen in humans (Carrier et al., 2011). Mechanistic aspects of such differences are unclear and will require further studies. It was demonstrated in human that a reduction of cortical thickness was responsible for an age-dependent reduction in slow wave activities (Dube et al., 2015). There is no difference in frontal cortical thickness between 1- and 3.5-month-old mice, but somatosensory cortex thickness decreases (Grand’Maison et al., 2013), and at later ages (12, 18 months) the decrease was minor (Hébert et al., 2013). This might explain the relative stability of delta power in the barrel cortex in our experiments.

### Wake–Sleep Cycle and Aging in Mice

Mice seemed to be a very attractive model to study aging. By the age of 3 months, they become mature adults, and at the age 10–15 months they belong to middle-aged group and after 18 months they are considered as being old (Flurkey et al., 2007). The aging in mice occurs substantially faster than in other laboratory animals and if the model is good, it would give multiple practical advances to study aging. Here we investigated several parameters of sleep–wake cycle in C57bL/6 mice in aiming to evaluate appropriateness of this mice strain to study in future the mechanistic aspects of human sleep.

During aging there are major alterations in human sleep. We took several appropriate parameters of human sleep affected by aging (Mander et al., 2017) and compared them to our results (1) with aging, humans have overall shorter sleep duration but some reports indicated that they sleep more during the day. *In our study*, the overall duration of SWS was higher in older mice, due longer SWS time only during the dark phase of the cycle (Figure 3D). Thus, while overall time spent in sleep is *different* between species, both show a *similar* increase of sleep during their active phase (light for humans, dark for mice). (2) *Similar* to human that reveal increased sleep fragmentation with aging, sleep fragmentation was much higher in older mice compared to young that is evidenced by a larger number of short states. (3) Older human have increased time spent awake throughout the night; however, older mice showed just a trend for increased time spent in a wake state throughout the light period, that is a period at which sleep dominates. (4) Older humans display an increased frequency of diurnal naps. In older mice, in the dark phase, there was not only an increase in the number of microarousals/micro sleep states, but also in the number of SWS episodes lasting between 10 and 60 s, which might be considered as “naps,” and there was an increased total duration of sleep, and is therefore similar to human. (5) Humans show an overall reduced slow-wave activity with aging. *Different* from human, in our mice experiments, the overall slow-wave activity did not undergo systematic changes. We found an increase with aging in the delta power and a trend for an increased slow wave density in frontal cortex, but stable delta power in the posterior part of somatosensory cortex. (6) Our data demonstrate that older mice showed a decrease in REM sleep, in particular at the end of dark phase and the beginning of the light phase of the light/dark cycle (Figure 2D). Although effects of aging in humans are more prominent in NREM sleep, reductions in REM sleep are also reported in healthy older participants (Carrier et al., 1997).

One aspect of our study investigated the spatial distribution of formally defined states over the cortical surface. We found that electrodes implanted in frontal cortex would identify more of wake, in motor and somatosensory cortex more SWS and in visual and retrosplenial cortex more REM sleep (Figure 6). REM sleep-related theta activity was more often seen in posterior parts of the dorsal cortical surface and slow-wave activity during otherwise REM sleep was often recorded over somatosensory and motor cortical areas (Figure 6). This supports a recent discovery of REM sleep-dependent slow wave activity in mice (Funk et al., 2016) except that we did not see REM sleep slow wave activities in visual cortex, and our recordings identified clear slow waves in layer 5, but not restricted to layer 4 as in the Funk et al.’s (2016) study. The observed dominance of wake, SWS, and REM sleep regions in our study largely overlaps with the anatomical localization of cortical clusters belonging to the default mode network identified in awake mice (Vanni et al., 2017) suggesting that on a daily scale, sleep and wake activities interact over large cortical areas. Two types of slow-wave activities during REM sleep were also found in human (Bernardi et al., 2019). Frontal–central slow waves, which authors believe are analogous with PGO waves, had increased gamma activity during slow waves (Bernardi et al., 2019, their Figure 4E). It is well-demonstrated that sleep slow waves in both human and animals are associated with a reduction of gamma activities (Mukovski et al., 2007; Cash et al., 2009; Csercsa et al., 2010; Bukhtiyarova et al., 2019). Some field potential deflections during REMs are expected because of synchronous increase in inhibitory activities in the cortex (Timofeev et al., 2001), which typically mediate gamma oscillations (Whittington et al., 2000; Buzsaki and Wang, 2012). Medial occipital slow waves although show a reduction in gamma power, but in the shown example (Bernardi et al., 2019, their Figure 7A), consist of an oscillation resembling spindle, not slow wave. It should be noted that a reduction in gamma frequency activities occurs more often than slow waves and often it cannot be used as a solo mean of slow wave detection (Bukhtiyarova et al., 2019). Therefore, the low frequency activity recorded during REM sleep by Bernardi et al. (2019) does not share the known feature of sleep slow waves.

## Conclusion

In this study we investigated the sleep–wake cycle and slow-wave activities of young and older mice during sleep. The ultimate goal was to evaluate the appropriateness of mice as a model to study human sleep during aging. We suggest that overall the aging mice sleep shares several common features human sleep. The major difference is an increase in older mice of the delta power in frontal cortex, while it decreases in human.

## Data Availability Statement

The datasets generated for this study are available on request to the corresponding author.

## Ethics Statement

All experiments were performed in accordance with the guideline of the Canadian Council on Animal Care and approved by the Université Laval Committee on Ethics and Animal Research.

## Author Contributions

SS, SC, and JS performed the experiments. SS, SC, and IT wrote the manuscript. SC, OB, J-ML, and JD developed the analytical tools. SS, SC, JS, OB, JD, and IT analyzed the data. JC contributed to the experimental design. IT supervised the experiments and analysis, and planned the research. All authors corrected and approved the final version of the manuscript.

## Conflict of Interest

The authors declare that the research was conducted in the absence of any commercial or financial relationships that could be construed as a potential conflict of interest.

## Supplementary Material

MOVIE S1 Dynamic of sleep–wake states detected in individual cortical electrodes based on delta power and theta power in each cortical electrode and neck muscle tone. The movie is played at a faster speed and the time is indicated in the top left corner.
